# Metabolomic Profiling of Amino Acids in Human Plasma Distinguishes Diabetic Kidney Disease From Type 2 Diabetes Mellitus

**DOI:** 10.3389/fmed.2021.765873

**Published:** 2021-11-29

**Authors:** Chunyu Zhou, Qing Zhang, Liqian Lu, Jiao Wang, Dongwei Liu, Zhangsuo Liu

**Affiliations:** ^1^Blood Purification Center, First Affiliated Hospital of Zhengzhou University, Zhengzhou, China; ^2^Research Institute of Nephrology, Zhengzhou University, Zhengzhou, China; ^3^Department of Nephrology, First Affiliated Hospital of Zhengzhou University, Zhengzhou, China; ^4^Key Laboratory of Precision Diagnosis and Treatment for Chronic Kidney Disease in Henan Province, Zhengzhou, China

**Keywords:** amino acid, metabolomics, UPLC-MS /MS, type 2 diabetes mellitus, diabetic kidney disease

## Abstract

**Background:** Diabetic kidney disease (DKD) is a highly prevalent complication in patients with type 2 diabetes mellitus (T2DM). Patients with DKD exhibit changes in plasma levels of amino acids (AAs) due to insulin resistance, reduced protein intake, and impaired renal transport of AAs. The role of AAs in distinguishing DKD from T2DM and healthy controls has yet to be elucidated. This study aimed to investigate the metabolomic profiling of AAs in the plasma of patients with DKD.

**Methods:** We established an ultra-performance liquid chromatography tandem mass spectrometry (UPLC-MS/MS) method to detect the plasma levels of the 20 AAs in healthy controls (*n* = 112), patients with T2DM (*n* = 101), and patients with DKD (*n* = 101). The key AAs associated with DKD were identified by orthogonal partial least-squares discriminant analysis (OPLS-DA) models with loading plots, shared and unique structures (SUS) plots, and variable importance in projection (VIP) values. The discrimination accuracies of these key AAs were then determined by analyses of receiver-operating characteristic (ROC) curves.

**Results:** Metabolomic profiling of plasma revealed significant alterations in levels of the 20 AAs in patients with DKD when compared to those in either patients with T2DM or healthy controls. Metabolomic profiling of the 20 AAs showed a visual separation of patients with DKD from patients with T2DM and healthy controls in OPLS-DA models. Based on loading plots, SUS plots, and VIP values in the OPLS-DA models, we identified valine and cysteine as potential contributors to the progression of DKD from patients with T2DM. Histidine was identified as a key mediator that could distinguish patients with DKD from healthy controls. Plasma levels of histidine and valine were decreased significantly in patients with DKD with a decline in kidney function, and had excellent performance in distinguishing patients with DKD from patients with T2DM and healthy controls according to ROC curves.

**Conclusion:** Plasma levels of histidine and valine were identified as the main AAs that can distinguish patients with DKD. Our findings provide new options for the prevention, treatment, and management of DKD.

## Introduction

Diabetic kidney disease (DKD) is a common complication of type 2 diabetes mellitus (T2DM) and the leading cause of chronic kidney disease (CKD) (1). DKD is associated with a high risk of end-stage renal disease, cardiovascular disease, infection, and death; the incidence of this condition has increased significantly over the past 20 years (2). Although both T2DM and DKD are related to hyperglycemia, it is incredibly challenging to predict the development of DKD by monitoring blood glucose since in clinical practice, many patients with DKD exhibit blood glucose levels that are similar to patients with T2DM alone. At present, DKD is diagnosed based on histological features after invasive renal biopsy. However, the harvested tissue may be inadequate for the diagnosis, and the biopsy procedure can be complicated by hematuria or perirenal hematoma (3). The lack of a suitable approach for early detection is a major barrier to the prevention and treatment of DKD. Therefore, there is a clear need to develop novel biomarkers of the onset and progression of DKD.

Amino acids (AAs) are substrates used for the synthesis of proteins and other molecules and for energy production (4). Previous research has reported changes in the plasma and urinary AA profiles of patients with CKD (5). The provision of nutritional supplements containing AAs to patients with CKD may improve their nutritional status, including body mass index, serum albumin level, and even survival (6). Furthermore, an increasing body of evidence has associated abnormal AA profiles with T2DM and a prediabetic state, including impaired glucose tolerance (IGT) (7, 8). Therefore, modulating AA intake could delay the development of kidney injury and other metabolic complications in patients with T2DM (9, 10). Some AAs appear to accelerate the progression of T2DM to DKD, whereas others may have a renoprotective role (11). However, the association of the 20 types of AAs in patients with T2DM and DKD has yet to be investigated in detail. In this study, we investigated the plasma profiles of the 20 AAs in patients with DKD, patients with T2DM, and healthy controls, using a method based on ultra-performance liquid chromatography tandem mass spectrometry (UPLC-MS/MS). Our results provide a basis for the early identification of DKD based on changes in plasma levels of key AAs, and can guide future studies on the etiology, pathophysiology, and treatment of DKD.

## Methods

### Study Design and Participants

This study was approved by the Ethics Committee of the First Affiliated Hospital of Zhengzhou University (2020-KY-363) and conformed to the Declaration of Helsinki 1964 and its later amendments. Written and informed consent was obtained from all participants. Patients with DKD (DKD group) and patients with T2DM (T2DM group) were recruited from the Department of Nephrology and Department of Endocrinology, respectively, while healthy controls (CON group) were recruited from the Department of Physical Examination, of the First Affiliated Hospital of Zhengzhou University. Plasma samples were collected in anticoagulant tubes containing ethylenediaminetetraacetic acid (EDTA) that remained after clinical laboratory testing.

The inclusion criteria for patients with DKD were as follows: (1) age between 18 and 75 years; (2) diagnosed with both T2DM and DKD at our hospital for more than 5 years according to the clinical diagnostic criteria set by the American Diabetes Association (ADA) (12, 13); and (3) not suffering from any other clinically diagnosed severe disease including but not limited to tumors, nervous, digestive, or mental disorders; or infectious diseases. The inclusion criteria for patients with T2DM were as follows: (1) age between 18 and 75 years; (2) diagnosed with T2DM for more than 5 years at our hospital according to the clinical diagnostic criteria set by the ADA; (3) not suffering from albuminuria and/or reduced estimated glomerular filtration rate (eGFR) in the absence of the signs or symptoms of other primary causes of kidney damage; and (4) not suffering from any other clinically diagnosed severe disease including but not limited to tumors, nervous, digestive, or mental disorders, or infectious diseases. The healthy controls were sex- and age-matched to the patients with DKD and patients with T2DM at a statistical significance level >0.05. The inclusion criteria for healthy controls were as follows: (1) age between 18 and 75 years; (2) no self-reported or clinical diagnostic history of any chronic severe disease, including but not limited to tumors, nervous, renal, digestive, or mental disorders, as well as an infectious disease within 3 months; and (3) not receiving any medical treatment in the 3 months prior to blood collection.

### Preparation of Solutions and Samples for Metabolomic Profiling

Stock solutions for the 20 AAs were prepared by dissolving standard compounds (MedChem Express, Monmouth Junction, NJ, United States) in water or dimethylsulfoxide at concentrations ranging from 1 to 100 mM and stored in brown volumetric flasks at −80°C until use. An isotope-labeled mix of 20 AAs mix (MilliporeSigma, Burlington, MA, United States) was diluted with acetonitrile (ACN, Thermo Fisher Scientific, Waltham, MA, United States) at a concentration of 100 nM to act as an internal standard (IS). Working solutions of AAs were prepared by diluting stock solutions into seven different batches (10–1,000 nM) with ACN.

Plasma samples and standard curves were prepared using a protein precipitation extraction method. In brief, 50 μl of thawed plasma and 50 μl of ACN were transferred to a 1.5-ml tube containing 150 μl of IS solution (100 nM). The standard curves were then generated by mixing 50 μl of plasma (mixed from 30 healthy controls), 50 μl of working solution, and 150 μl of IS. All samples underwent vortex-mixing for 10 min, followed by centrifugation at 15,000 rpm for 10 min at 4°C. The supernatant was transferred into a 250-μl insert fixed in a 2-ml capped vial (Agilent Technologies, Santa Clara, CA, United States) for further UPLC-MS/MS detection.

### Detection of AAs in Plasma Samples Using UPLC-MS/MS

Amino acids were detected by UPLC-MS/MS in positive-ion multiple reaction monitoring mode on a UPLC-30ADvp series instrument (Shimadzu, Kyoto, Japan) with an SIL-30-AC autosampler, CTO-20 AC column oven, and an API 6500 triple-quadrupole source (Applied Biosystems Sciex, Toronto, ON, Canada). [M+H]^+^ precursor ions were used for AAs and ISs. The optimized MS parameters for compounds were as follows: capillary voltage, 5,500 V; source temperature, 550°C; curtain gas (N_2_), 30 psi; collision gas, 10 psi; pressure for nebulization gas, 40 psi; pressure for evaporation gas, 40 psi; entrance potential, 50 V; and collision cell exit, 10 V. The detailed declustering potential and collision energy, as well as the precursor and dominant daughter ions of the AAs and ISs, are listed in Supplementary Tables 1, 2. Chromatographic separation was undertaken at 40°C using an UPLC BEH amide column (2.1 × 100 mm, 3-μm particle size; Waters, Milford, MA, United States) equipped with a 1.7-μm VanGuard precolumn; the isocratic gradient elution program (Supplementary Table 3) was run with mobile phase A (water containing 0.1% formic acid and 0.05% trifluoroacetic acid) and mobile phase B (ACN). Analyst v1.6.2 software was used for data acquisition and processing. The chromatograms of AAs and isotope-labeled AAs were conducted by OriginLab (OriginLab, Northampton, MA, United States).

### Metabolomic Profiling of Plasma AAs

Plasma levels of AAs were imported into SIMCA-P v16.0.2 (Umetrics Suite; Sartorius, Umeå, Sweden). Orthogonal partial-least-squares discriminant analysis (OPLS-DA) was then used to explore the discrimination between patients with DKD, patients with T2DM, and healthy controls based on plasma levels of AAs. A loading plot was established to visualize the OPLS-DA model, and a shared and unique structures (SUS) plot was generated to determine the relative contributions of AAs to the group discrimination. The quality of all OPLS-DA models was evaluated by the goodness-of-fit parameter (R^2^) and the predictive ability parameter (Q^2^). A permutation plot was built to indicate the correlation coefficient between the original R^2^, original Q^2^, cumulative R^2^, and cumulative Q^2^. The variable importance in projection (VIP) value of the AAs in the model was also calculated to indicate its relative contribution to group discrimination. VIP value >1.1 was considered statistically significant in terms of discriminating between groups.

### Statistical Analysis

Continuous variables were expressed as mean ± SD and analyzed using Student's *t*-test, chi-squared test, and Fisher's exact test using Prism v8.0.2 software (GraphPad, San Diego, CA, United States). Receiver-operating characteristic (ROC) curves were performed with SPSS v21.0 (Armonk, NY, United States). The corresponding area under the ROC curve (AUC), cutoff value, sensitivity, specificity, and accuracy were conducted to evaluate the predictive performances of the key AAs for DKD. The accuracy was calculated using the equation:

(A × sensitivity + B × specificity)/(A + B)

where A is the participant number of the corresponding disease group, and B is the participant number of the corresponding control group. *P* < 0.05 was considered statistically significant.

## Results

### Plasma Profiles of the 20 AAs Distinguished Patients With DKD From Patients With T2DM and Healthy Controls

**Figure 1:**
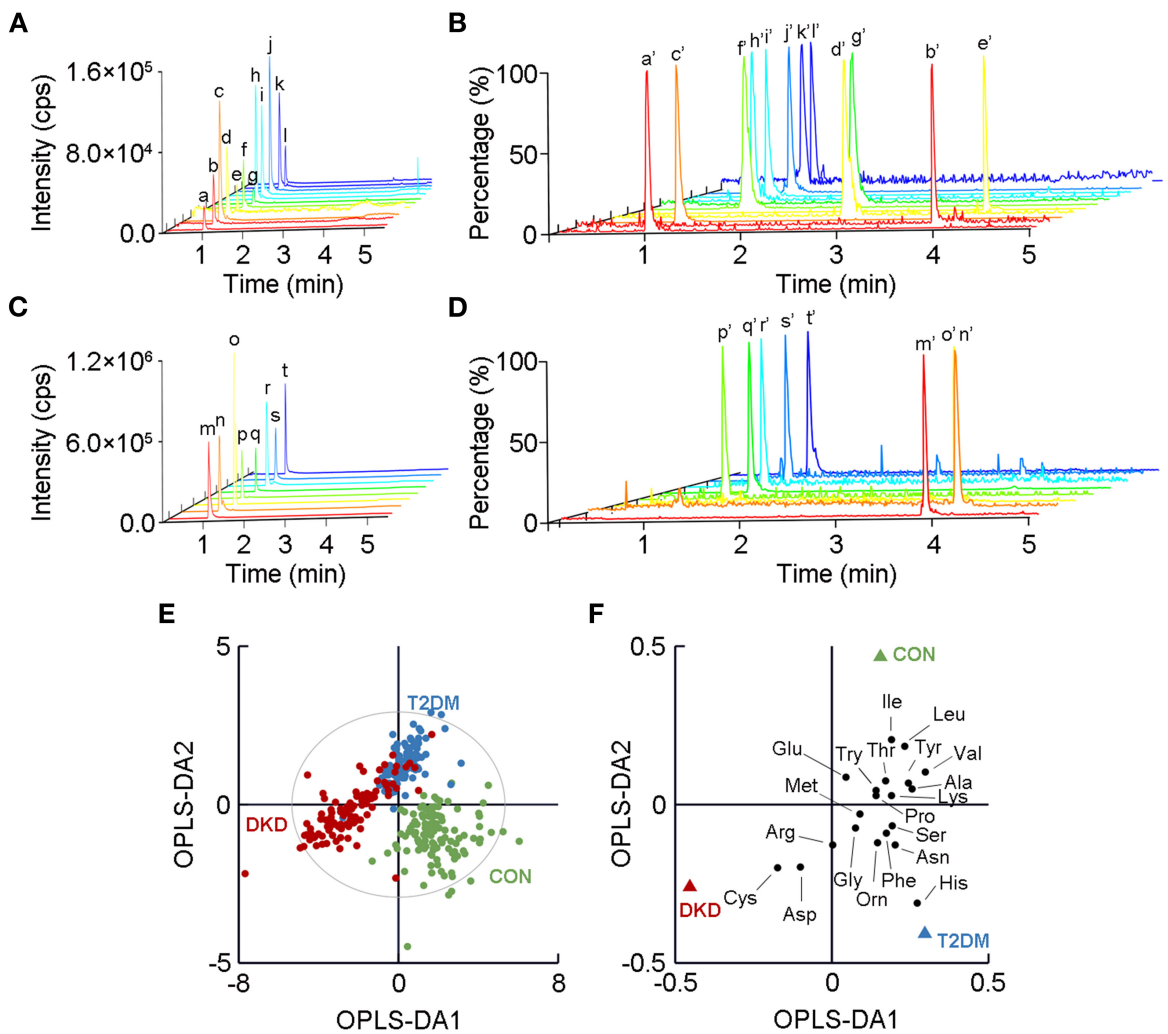
Metabolomic profiles of the 20 amino acids (AAs) in plasma in the DKD group, T2DM group, and CON group. A chromatogram of **(A)** low-response AAs and **(B)** their corresponding isotope-labeled internal standards (ISs) detected by ultra-performance liquid chromatography tandem mass spectrometry. A chromatogram of **(C)** high-response AAs and **(D)** their corresponding isotope-labeled ISs. *a* to *t* show mass-spectrometry plots for the 20 AAs, while *a'* to *t'* show the corresponding isotope-labeled ISs. Detailed information relating to *a* to *t* and *a'* to *t'* is listed in Supplementary Tables 1, 2. **(E)** Orthogonal partial-least-squares discriminant analysis (OPLS-DA) score plot shows the visual separation of the DKD group, T2DM group, and CON group. The ellipse indicates the Hotelling T2 (0.95) range for the model. **(F)** Loading plot analysis showing the relative contributions of the 20 AAs to the differences between the DKD, T2DM, and CON groups. DKD, diabetic kidney disease; T2DM, type 2 diabetes mellitus; CON, healthy control.

Blood samples from the CON, T2DM, and DKD groups were collected from October 2019 to September 2020. Eighty-two of 101 patients with DKD had been pathologically diagnosed with DKD by invasive renal biopsy previously. The demographic characteristics of the study population are summarized in Supplementary Table 4. We established a UPLC-MS/MS method to quantify the plasma levels of the 20 AAs (Figures 1A–D). These analyses revealed significant alterations of the 20 AAs in the DKD group when compared with those in the T2DM group and CON group (Table 1). We also analyzed the association between 20 AAs and difference in kidney function of patients with DKD. A total of 101 patients with DKD were divided into three groups according to CKD stages: stage 1 (eGFR ≥90 ml/min/1.73m^2^, *n* = 13), stages 2–3 (30 ml/min/1.73 m^2^ ≤ eGFR ≤ 89 ml/min/1.73 m^2^, *n* = 30), and stages 4–5 (eGFR ≤ 29 ml/min/1.73 m^2^ without renal replacement therapy, *n* = 58). Seven of 20 AAs decreased significantly with a decline in kidney function (Supplementary Table 5).

**Table 1:** Plasma levels of the 20 amino acids in study participants.

**AA (μM)**	**CON (*n* = 112)**	**T2DM (*n* = 101)**	**DKD (*n* = 101)**	**DKD vs. T2DM**		**DKD vs. CON**	
				**VIP**	***P***	**VIP**	***P***
Gly	303.3 ± 179.4 (97.0–1034)	232.6 ± 127.6 (59.3–775.9)	222.0 ± 157.3 (14.9–932.1)	0.717	0.600	0.801	<0.001
Ala	272.4 ± 104.7 (0.0–660.0)	238.8 ± 100.6 (63.0–598.1)	127.3 ± 80.1 (18.3–344.8)	1.266	<0.001	1.262	<0.001
Arg	286.6 ± 251.7 (22.3–1466)	206.6 ± 83.5 (52.3–498.4)	252.7 ± 126.6 (80.6–889.0)	0.657	0.003	0.657	0.223
Asn	49.3 ± 28.2 (8.8–144.4)	28.8 ± 16.8 (5.0–109.9)	20.2 ± 15.9 (1.4–69.0)	0.884	<0.001	1.157	<0.001
Asp	43.2 ± 52.7 (0.0–316.3)	18.0 ± 16.0 (0.0–129.3)	54.1 ± 64.4 (0.0–325.4)	0.992	<0.001	0.502	0.175
Cys	286.2 ± 165.5 (93.8–797.4)	200.2 ± 83.4 (90.0–456.9)	387.1 ± 210.4 (0.0–1056)	1.200	<0.001	0.700	<0.001
Glu	231.9 ± 133.6 (65.7–673.5)	242.7 ± 68.4 (99.1–495.7)	207.9 ± 90.8 (74.0–738.1)	0.572	0.002	0.621	0.1313
His	101.3 ± 30.6 (38.0–204.2)	21.4 ± 12.9 (4.6–81.9)	15.4 ± 14.0 (1.5–107.8)	0.740	0.002	1.593	<0.001
Iso	79.5 ± 19.3 (40.6–154.1)	89.2 ± 22.9 (47.8–163.8)	58.5 ± 27.9 (13.9–163.8)	1.233	<0.001	1.011	<0.001
Leu	146.2 ± 36.4 (64.0–239.2)	164.3 ± 38.4 (100.8–252.2)	93.0 ± 62.8 (10.7–297.4)	1.350	<0.001	1.091	<0.001
Lys	143.9 ± 54.3 (60.1–304.4)	126.5 ± 51.2 (39.6–285.6)	86.6 ± 66.5 (5.9–274.8)	1.086	<0.001	1.046	<0.001
Met	59.2 ± 17.8 (21.9–167.6)	53.7 ± 16.0 (27.4–112.1)	49.0 ± 19.0 (14.4–143.3)	0.783	0.057	0.760	<0.001
Orn	59.4 ± 37.3 (15.3–281.8)	36.4 ± 16.8 (13.2–132.5)	34.0 ± 21.9 (3.1–152.5)	0.770	0.390	0.945	<0.001
Phe	87.6 ± 23.3 (50.2–162.7)	72.8 ± 13.2 (44.3–126.1)	65.3 ± 24.8 (24.5–127.2)	0.861	0.007	1.017	<0.001
Pro	157.8 ± 67.8 (55.4–587.3)	142.4 ± 53.7 (47.0–330.8)	106.2 ± 83.6 (7.8–407.3)	0.888	<0.001	0.840	<0.001
Ser	74.5 ± 34.3 (26.9–272.4)	56.8 ± 25.3 (13.0–156.0)	39.8 ± 26.7 (5.4–134.2)	1.003	<0.001	1.130	<0.001
Thr	88.7 ± 41.1 (0.3–197.7)	84.5 ± 36.3 (14.2–179.4)	54.4 ± 40.8 (4.3–190.2)	1.030	<0.001	0.887	<0.001
Trp	77.7 ± 23.4 (32.1–170.0)	71.9 ± 18.2 (20.0–119.6)	57.4 ± 33.5 (1.8–211.2)	0.756	<0.001	0.764	<0.001
Tyr	52.7 ± 14.3 (24.2–99.4)	49.4 ± 13.8 (16.7–106.7)	32.1 ± 14.3 (10.2–79.7)	1.181	<0.001	1.187	<0.001
Val	129.7 ± 38.2 (67.6–236.0)	121.7 ± 27.9 (62.8–210.1)	57.5 ± 46.1 (2.2–170.3)	1.442	<0.001	1.326	<0.001

Data represent mean ± SD (range). ^*^P-values were determined by the Student's t-test. μM, micromole; CON, healthy controls; T2DM, type 2 diabetes mellitus; DKD, diabetic kidney disease; Gly, glycine; Ala, alanine; Arg, arginine; Asn, asparagine; Asp, aspartic acid; Cys, cysteine; Glu, glutamic acid; His, histidine; Iso, isoleucine; Leu, leucine; Lys, lysine; Met, methionine; Orn, ornithine; Phe, phenylalanine; Pro, proline; Ser, serine; Thr, threonine; Try, tryptophan; Tyr, tyrosine; Val, valine.

Next, we used OPLS-DA, a supervised multivariate statistical method, to highlight the visual separation among the DKD, T2DM, and CON groups (R^2^X = 0.699, R^2^Y = 0.650, Q^2^ = 0.614; Figure 1E). This result was validated by the random permutation test (Supplementary Figure 1). More specifically, 17 of the AAs showed differences in levels upon a comparison between the DKD group and the T2DM group, whereas 18 of the 20 AAs showed differences in levels upon a comparison between the DKD group and the CON group. Among the detected AAs, histidine, valine, and cysteine showed the greatest differences among the three groups in terms of their levels (Figure 1F).

### Plasma Levels of Valine and Cysteine Revealed Them to Be the Major AAs Contributing to the Group Difference Between Patients With DKD and Patients With T2DM

**Figure 2:**
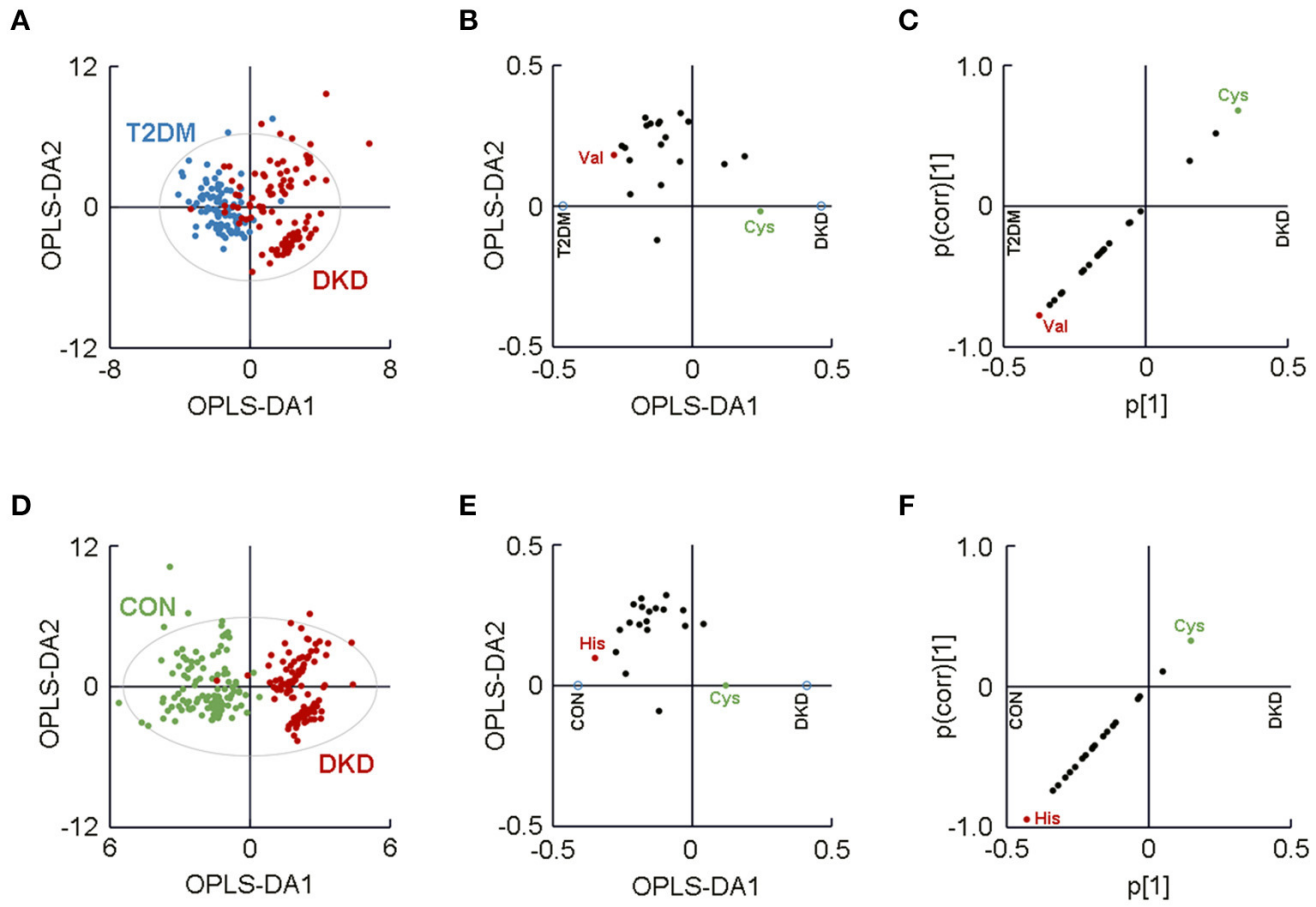
Separated metabolomic analyses highlighted visual separation of the DKD group from the T2DM group and the CON group. **(A)** Orthogonal partial-least-squares discriminant analysis (OPLS-DA) plot showing the visual separation between the DKD group and the T2DM group. The **(B)** loading plot and **(C)** SUS plot identified plasma levels of valine and cysteine to be the major contributors to the separation between the DKD group and the T2DM group. **(D)** OPLS-DA plot showing the visual separation between the patients with DKD and CON group. The **(E)** loading plot and **(F)** SUS plot identified plasma levels of histidine and cysteine as the major amino acids contributing to the difference between the DKD group and the CON group. The ellipse indicates the Hotelling T2 (0.95) range for the model. DKD, diabetic kidney disease; T2DM, type 2 diabetes mellitus; CON, healthy control.

The separated OPLS-DA score plot enabled the visualization of the separation between the DKD group and T2DM group (R^2^X = 0.632, R^2^Y = 0.610, Q^2^ = 0.563; Figure 2A). Then, we generated a loading plot and SUS plot based on the OPLS-DA model to assess the relative contributions of the 20 AAs to the separation between groups (Figures 2B,C). Plasma levels of valine and cysteine were identified as the major AAs that contributed to the difference between the DKD group and the CON group. In addition, the VIP values for valine and cysteine were greater than 1.10 (Table 1), which highlighted the significant contribution of these two AAs to the group difference. Collectively, our metabolomic profiling demonstrated valine and cysteine as the major AAs that permitted discrimination of patients with DKD from patients with T2DM.

### Plasma Levels of Histidine Revealed It to Be a Major AA Contributing to the Group Difference Between Patients With DKD and Healthy Controls

Another separated OPLS-DA score plot was generated to highlight the visual separation between the DKD group and the CON group (R^2^X = 0.682, R^2^Y = 0.817, Q^2^ = 0.778; Figure 2D). Histidine and cysteine were identified as the major contributors to the difference between the DKD group and the CON group, as determined by a loading plot and SUS plot (Figures 2E,F). The VIP value of histidine was 1.593 (>1.10) yet the VIP value for cysteine was 0.700. Therefore, these metabolomic analyses indicated that histidine was the major AA that could be used to distinguish between patients with DKD and healthy controls.

### Plasma Levels of Histidine and Valine Had an Excellent Predictive Ability to Separate Patients With DKD From Patients With T2DM and Healthy Controls

**Figure 3:**
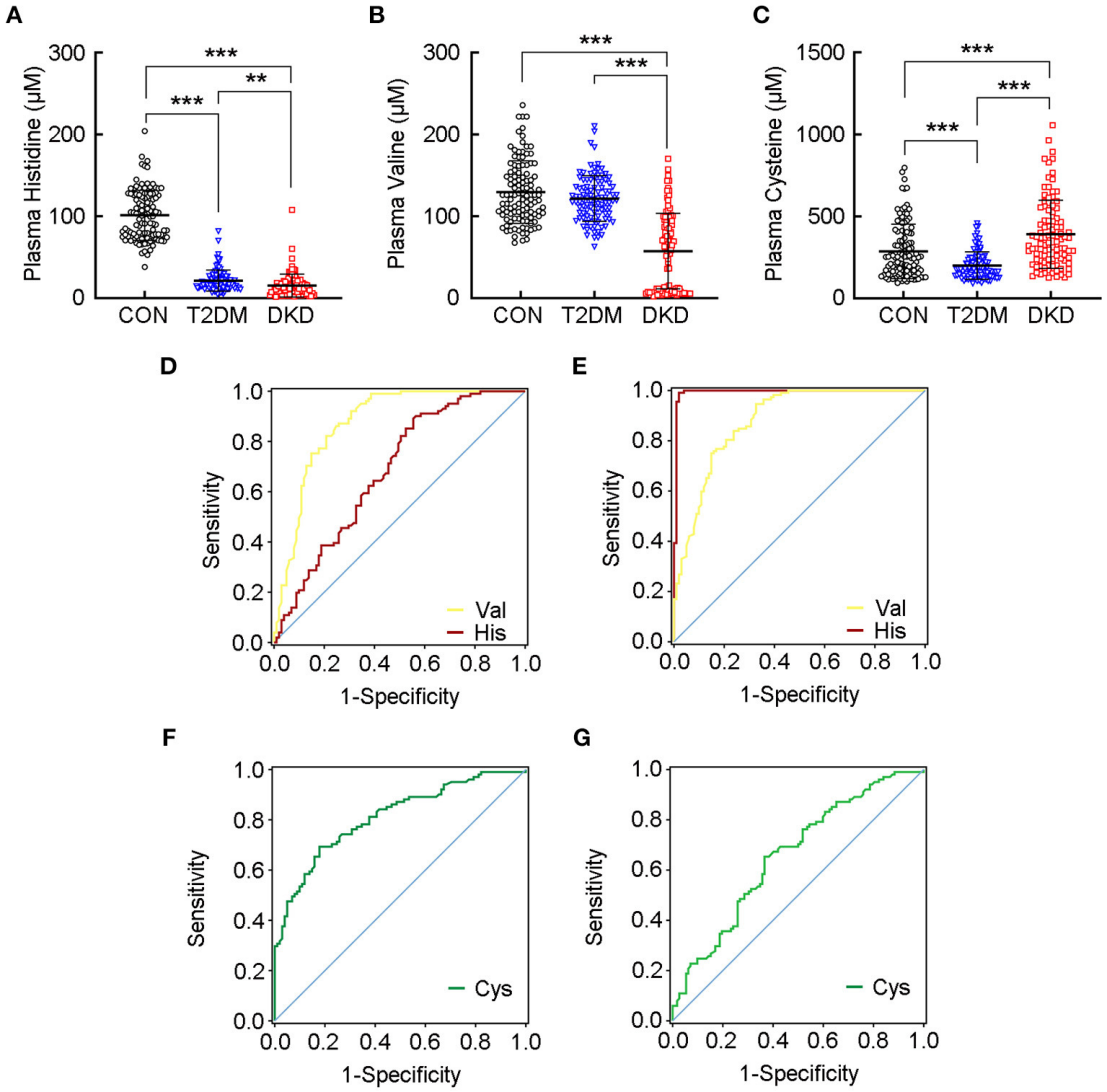
Plasma levels of histidine and valine have an excellent predictive ability to separate patients with DKD from patients with T2DM and healthy controls. Plasma levels of **(A)** histidine, **(B)** valine, and **(C)** cysteine, in the CON group, T2DM group, and DKD group, respectively. Receiver-operating characteristic (ROC) curve demonstrates that the plasma level of valine can discriminate patients with DKD from patients with T2DM **(D)**, and from healthy controls **(E)** according to area under the ROC curve (AUC) values greater than 0.85, whereas the plasma level of histidine has excellent predictive ability to discriminate patients with DKD from healthy controls **(E)**. ROC curve shows much lower AUC values for cysteine in both patients with DKD vs. patients with T2DM **(F)**, and patients with DKD vs. healthy controls **(G)**. Detailed AUC values are presented in Supplementary Table 6. **P < 0.01, ***P < 0.001. CON, healthy control; DKD, diabetic kidney disease; T2DM, type 2 diabetes mellitus.

When analyzing the plasma levels of histidine, valine, and cysteine, we observed a significant reduction in the levels of histidine in the DKD group and T2DM group compared with that in the CON group (*P* < 0.001; Figure 3A). We also identified a reduction in the plasma levels of valine in the DKD group compared with that in the T2DM group or the CON group (*P* < 0.001; Figure 3B). In addition, the plasma level of cysteine was reduced in the T2DM group compared with that in the CON group, but was higher in the DKD group than that in the CON group and T2DM group (*P* < 0.001; Figure 3C).

Next, we explored the plasma levels of the 20 AAs in DKD patients with different levels of kidney function (Supplementary Table 5) and found that the plasma level of histidine was decreased in CKD stages 4–5 compared with that in stage 1 or stages 2–3. The plasma level of valine was decreased significantly in CKD stages 2–3 compared with that in stage 1, and much lower in stages 4–5. These results indicated that the plasma levels of histidine and valine were decreased significantly with a decline in kidney function.

Next, we constructed ROC curves for the plasma levels of histidine, valine, and cysteine to evaluate their predictive performance to separate the DKD group from the T2DM group or CON group (Table 2; Supplementary Table 6). Valine had an AUC values greater than 0.85 for DKD vs. T2DM (AUC = 0.874 ± 0.025, *P* < 0.001; Figure 3D) and DKD vs. CON (AUC = 0.881 ± 0.023, *P* < 0.001; Figure 3E). Further analyses indicated the excellent sensitivity of valine for the predictive performance of the DKD group compared with that of the T2DM group (sensitivity = 92.08%, specificity = 69.31%, accuracy = 80.70%) and CON group (sensitivity = 94.64%, specificity = 67.33%, accuracy = 80.81%). The AUC value for histidine was much greater than 0.90 for DKD vs. CON (AUC = 0.993 ± 0.006, *P* < 0.001; Figure 3E), but was lower for DKD vs. T2DM (AUC = 0.681 ± 0.038, *P* < 0.001; Figure 3D). The sensitivity, specificity, and accuracy of histidine in DKD vs. CON group were much greater than 95%. In addition, the AUC for cysteine was much lower for DKD vs. T2DM (AUC = 0.811 ± 0.030, *P* < 0.001; Figure 3F) and DKD vs. CON (AUC = 0.659 ± 0.037, *P* < 0.001; Figure 3G) with a corresponding lower sensitivity, specificity, and accuracy. These results suggested that the plasma levels of histidine and valine are key markers for distinguishing patients with DKD from patients with T2DM and healthy controls.

**Table 2:** Predictive performance of the key amino acids.

	**AUC**	*P* ^*^	**Cutoff value (μM)**	**Sensitivity (%)**	**Specificity (%)**	**Accuracy (%)**
**DKD vs. T2DM**						
His	0.681 ± 0.038	<0.001	10.7	90.10	43.56	66.83
Val	0.874 ± 0.025	<0.001	85.8	92.08	69.31	80.70
Cys	0.189 ± 0.030	<0.001	259.4	69.31	82.18	75.75
**DKD vs. CON**						
His	0.993 ± 0.006	<0.001	50.6	99.11	98.02	98.53
Val	0.881 ± 0.023	<0.001	81.4	94.64	67.33	80.81
Cys	0.341 ± 0.037	<0.001	287.4	65.36	63.39	64.32

* *P values were determined by analyses of ROC curves under a nonparametric assumption. The AUC values of all 20 AAs are presented in Supplementary Table 6. Accuracy = (A × sensitivity + B × specificity)/(A + B), where A is the participant number of the corresponding disease group and B is the participant number of the corresponding control group. ROC, receiver-operating characteristic; AUC, area under the ROC curve; His, histidine; Val, valine; Cys, cysteine*.

## Discussion

Using a UPLC-MS/MS-based metabolomic approach, we demonstrated, for the first time, that the plasma profiles of the 20 AAs could be used to distinguish patients with DKD from patients with T2DM and healthy controls. Supervised OPLS-DA models were established to evaluate the visual separation of patients with DKD from patients with T2DM and healthy controls. The R^2^ values of each OPLS-DA model indicated the corresponding goodness of fit, whereas Q^2^ values indicated that the OPLS-DA models we used for analyses were valid. Further metabolomic analyses showed that valine and cysteine may be the major AAs that contribute to the group differences between the DKD group and the T2DM group. Histidine was identified as a major contributor to the group difference between the DKD group and the CON group. ROC curves provided further validation that the decreased plasma levels of histidine and valine are key markers for identifying patients with DKD. These reductions were strongly associated with a decline in kidney function. Taken together, our study revealed that the reduced plasma levels of histidine and valine could be used to distinguish patients with DKD from patients with T2DM and healthy controls.

Amino acids have important roles in multiple pathophysiological processes, including the mediation of blood glucose. Disturbances in AA metabolism are closely implicated in the pathogenesis of T2DM (14). Increased plasma levels of branched-chain amino acids (BCAAs), including leucine, isoleucine, and valine, aromatic amino acids (AAAs), methionine, and glutamate-to-glutamine ratios, are associated significantly with T2DM (15–17). This positive association can also be detected prior to the onset of T2DM, even in prediabetes or obesity (7, 18). Emerging evidence attributes these alterations mainly to insulin resistance (IR) rather than insufficient secretion of insulin (19). Four possible mechanisms underlie these associations. First, an increased plasma level of BCAAs leads to hyperactivation of the mammalian target of rapamycin (mTOR) signaling pathway, thereby resulting in the dysfunction and destruction of pancreatic beta-cells (20). Second, BCAAs may facilitate glucose uptake, as well as glycogen synthesis, *via* the phosphatidylinositol 3-kinase (PI3K) or protein kinase C (PKC) signaling pathways, in an insulin-independent manner (21). Third, inflammation and endoplasmic reticulum stress inhibit the expression of BCAA metabolic enzymes in white adipose tissue, thus resulting in BCAA accumulation (14, 22). Fourth, enrichment of BCAA-producing and AA-degrading bacterial species in the intestine are important environmental factors that contribute to the progression of T2DM (23, 24). In contrast with those findings, we discovered that the levels of 13 of the 20 AAs were reduced significantly in the plasma of patients with T2DM compared with those in healthy controls; only plasma levels of isoleucine and leucine were increased. These conflicting results indicate that the etiology of T2DM, and its association with AAs, is complex and poorly understood. It is likely that previous studies may have involved confounding factors, including species, the types of supplemented or restricted AAs, and the route of AA administration (25–28). Furthermore, it is possible that IR extends beyond glucose metabolism to lipid metabolism and energy metabolism (14, 29).

Increased levels of glucose can activate several signaling pathways in various tissues. This circumstance can arise because of dysfunction of pancreatic beta-cells or IR, or hyperglycemia-induced oxidative stress with the formation of reactive oxygen species (ROS), reactive carbonyl species (RCS), and advanced glycation end products (AGEs) (30). Oxidative stress causes IR, IGT, and endothelial dysfunction in T2DM and represents a fundamental factor underlying the pathogenesis of micro- and macrovascular complications (31). DKD is a devastating vascular complication in patients with T2DM and is characterized histopathologically by thickening of the glomerular basement membrane, mesangial expansion, nodular glomerular sclerosis, and even tubulointerstitial fibrosis (32). ROS (e.g., hydrogen peroxide) generation is the main factor that leads to posttranslational modifications due to AA oxidation. RCS (e.g., methylglyoxal) react with arginine residues to form the main substrates for AGEs. The formation of methylglyoxal-derived AGEs and methylglyoxal-derived hydroimidazolone are correlated significantly with DKD development (33). Our present findings, and those of other studies, indicate that AA metabolism is strongly associated with the pathogenesis of DKD.

Aside from IR or oxidative stress, another factor that accounts for the lower AA levels in patients with DKD is the impaired transport of AAs in the kidney and intestine. This action results in an inadequate supply of AAs to all tissues and the disruption of AA homeostasis in plasma (4). The solute carrier (SLC) superfamily is the largest protein family of amino acid transporters (AATs) (34). SLC6A19 is the major AA transporter and is expressed predominantly in the S3 segments of the proximal renal tubule, thus facilitating the transport of a broad range of AAs. Impairment of the AA transport systems has been reported to lead to the dysfunction of AA reabsorption in a hyperglycemic environment (35). Furthermore, uremic toxins in patients with CKD can lead to alterations of the intestinal flora that produces gut-derived toxins (36). These gut-derived toxins, together with uremic toxins, can impair AA transport in both the intestine and the proximal renal tubules, thus resulting in alterations in plasma levels of AAs (37, 38). In addition, nutrition can interfere with DKD progression. The dietary protein requirements of T2DM patients without kidney injury are considered to be the same as those of the general population. In DKD however, a low-dietary-protein diet (LDP) is recommended; this is another factor that accounts for the lower AA levels (39). LDP, in conjunction with essential AAs and ketoacids, may reduce the extent of proteinuria, uremic burden, metabolic derangements, and renal oxidative stress, thereby leading to retardation in the progression of kidney injury (40).

Our metabolomic analysis demonstrated that the plasma level of histidine was reduced significantly in patients with DKD. Histidine exerts anti-inflammatory and antioxidant effects. These effects are based on a combination of free-radical elimination and metal chelation (41). Insufficient levels of plasma histidine have been shown to be associated with persistent inflammation and oxidative stress in renal diseases, which can be relieved by the dietary supplementation with histidine (42, 43). This association has been reported to be strongly associated with histidine metabolism (44). Carnosine is the major endogenous histidine-containing dipeptide (HDP) and has a renoprotective role in T2DM and DKD. Sauerhofer and colleagues showed that insulin secretion could be preserved by increasing carnosine levels; this treatment increased the mass of pancreatic beta-cells (45). In diabetes, reduced levels of carnosine would attenuate the renoprotective role of HDPs. Oral carnosine supplementation could improve glucose tolerance and alleviate structural and functional renal damage *via* anti-inflammatory, antioxidant, anti-glycation, and reactive carbonyl-quenching mechanisms (46, 47). Anserine is another form of HDP that is obtained exogenously from protein supplementation. Anserine can be synthesized by carnosine methylation in most mammals, fish, and amphibians, but not in humans (48). Anserine exerts the same effects as carnosine and can alleviate glucose metabolism, proteinuria, and vascular permeability under diabetic conditions by inhibiting glycation, reducing oxidative damage, and enhancing antioxidant activity (49). However, anserine has a higher level of antioxidative activity than carnosine, which can activate the intracellular heat shock protein 70 /heme oxygenase 1 defense system under oxidative and glycative stress (50). Therefore, there is strong evidence that abnormal metabolism of histidine can contribute to the development of DKD from T2DM. The reduced plasma levels of histidine in patients with T2DM and patients with DKD possibly attribute to the enhanced histidine metabolism, including HDPs hydrolysis and histidine methylation, in diabetic conditions.

Our study observed a significant decrease in the plasma level of valine in patients with DKD than in patients with T2DM and healthy controls. This decrease was much more severe with the progression of DKD, which was also reported by Esmati and colleagues (51). Valine undergoes transamination by branched-chain aminotransferases in mitochondria to generate 3-methyl-2-oxobutyrate. The latter is dehydrolyzed by the branched-chain ketoacid dehydrogenase complex, which then produces isobutyryl-coenzyme A, a raw material for the odd-chain fatty acid (OCFA) synthesis. Intake of dairy proteins and fats is thought to be the major reason for changes in the circulating level of valine and its metabolites (52). Valine contributes to the increased levels of OCFAs in the liver and the circulation in patients with T2DM *via two* distinct mechanisms: increased α-oxidation and d*e novo* OCFA lipogenesis (53). In addition, Xiao and coworkers showed that valine deprivation may have a beneficial role in the prevention of sensitivity to glucose and insulin in mice and that this process was mediated by reduced mTOR/S6K1 signaling, and increased AMP-activated protein kinase (AMPK) signaling (54). However, as the plasma level of valine was not altered significantly in patients with T2DM, there might be some other factors contributing to its specific decrease in DKD other than the progression of the diabetic condition.

Studies have shown that the AA profiles of patients with early CKD are not altered significantly from those of healthy controls, whereas those were differed by diabetes in the early stage of CKD (55). Lee et al. showed that the plasma level of valine was lower in the CKD patients with T2DM than in people not suffering from T2DM, which reflects the substantially high risk associated with the diabetic condition (56). They also indicated that the effects of diabetic condition on the altered AA metabolism were greater in the early stage rather than in advanced CKD. Gabbai and colleagues reported that a hyperglycemic environment leads to the impairment of the renal AA transport systems even in the normal kidney (6). Prasad et al. reported excessive excretion of AAs owning to the glomerular hyperfiltration in the early stage of DKD, but was released in the advanced CKD (57). Those results indicated that the metabolic alterations of AAs from the diabetic condition were more substantial than those from the renal impairment in the early stage of CKD. In advanced CKD however, renal impairment was the dominant factor contributing to the AA alterations (5, 58). In accordance with those findings, we discovered that the plasma levels of seven of 20 AAs, including histidine and valine, were decreased significantly in DKD patients with a decline in kidney function. Thus, these reductions in patients with DKD were not only merely a result of diabetes progression, but a consequence of a decline in kidney function as well.

Some limitations to our study need to be considered. First, the sample size was relatively small; therefore, we could not sufficiently determine the plasma profiles of the 20 AAs at different stages of DKD. Second, we described the preliminary association between DKD and the plasma levels of histidine or valine, but we did not determine the specific mechanisms involved. Third, as this was a cross-sectional study, the causal relationship between T2DM, DKD, and AA alterations could not be determined. Fourth, we could not identify whether our findings were a result of a decrease in kidney function or a result of the DKD progression from T2DM because the sample sizes varied widely between patients with T2DM (T2DM group, *n* = 101) and patients with albuminuria and normal eGFR (CKD stage 1 in the DKD group, *n* = 13), which results in little performance of statistic test. Therefore, further investigations, including the prospective longitudinal study, *in_vivo*, or *in_vitro* intervention studies, are needed to explore the association between DKD development and the metabolic activities of histidine and valine.

## Conclusion

Our study demonstrated that the decreased plasma levels of histidine and valine could be considered as potential markers to distinguish patients with DKD patients from patients with T2DM and healthy controls. Although further mechanistic studies are required, our findings may have clinical relevance for the early diagnosis and treatment of DKD.

## Data Availability Statement

The raw data supporting the conclusions of this article will be made available by the authors, without undue reservation.

## Ethics Statement

The studies involving human participants were reviewed and approved by the Ethics Committee of the First Affiliated Hospital of Zhengzhou University (2020-KY-363). The patients/participants provided their written informed consent to participate in this study.

## Author Contributions

CZ and QZ designed the study, analyzed the data, and wrote the manuscript. QZ, LL, and JW carried out the experiments. CZ, QZ, DL, and ZL finalized the manuscript. DL and ZL supervised the study. All authors reviewed and approved the final version of the manuscript.

## Funding

This study was supported by grants from the National Natural Science Foundation of China (81970633) and the Comprehensive and Digital Demonstration Platform for Clinical Evaluation Technology of New Drugs for Major Diseases (2020ZX09201-009).

## Conflict of Interest

The authors declare that the research was conducted in the absence of any commercial or financial relationships that could be construed as a potential conflict of interest.

## Publisher's Note

All claims expressed in this article are solely those of the authors and do not necessarily represent those of their affiliated organizations, or those of the publisher, the editors and the reviewers. Any product that may be evaluated in this article, or claim that may be made by its manufacturer, is not guaranteed or endorsed by the publisher.
