# Supplementary material for: Metabolomic Profiling of Amino Acids in Human Plasma Distinguishes Diabetic Kidney Disease From Type 2 Diabetes Mellitus
Source: Front Med (Lausanne). 2021 Nov 29;8:765873. doi: 10.3389/fmed.2021.765873 (PMC8666657; doi:10.3389/fmed.2021.765873)
Supplement: Supplementary file 1 [file Data_Sheet_1.docx]

**Supplementary materials for**

## Metabolomic Profiling of Amino Acids in Human Plasma Distinguishes Diabetic Kidney Disease from Type 2 Diabetes Mellitus

**Table of Contents**

**Supplementary Table 1.** Mass spectrometry parameters, measurement range, and quantification of 20 amino acids

**Supplementary Table 2.** Mass spectrometry parameters of 20 isotope-labeled amino acids

**Supplementary Table 3.** Gradient program of liquid chromatography for 20 amino acids

**Supplementary Table 4.** General characteristics of the study population

**Supplementary Table 5.** Plasma levels of 20 amino acids in diabetic kidney disease patients with different levels of kidney function

**Supplementary Table 6.** Area under the receiver operating characteristic curve values for 20 amino acids in plasma of study participants

**Supplementary Figure 1.** Permutation plots of orthogonal partial least-squares discriminant models

**Supplementary Table 1.** Mass spectrometry parameters, measurement range, and quantification of 20 amino acids

| **Amino acid** | **MS no.^*^** | **Abbreviation** | **ID** | **CAS no.** | **Catalog ID^#^** | **MRM transition** | **DP**  **(V)** | **CE**  **(V)** | **Range**  **(nM)** | **Linearity (R^2^)** |
| --- | --- | --- | --- | --- | --- | --- | --- | --- | --- | --- |
| Glycine | a | Gly | HMDB0000123 | 56-40-6 | HY-N0390 | 76→30 | 20 | 18 | 10-1000 | 0.9985 |
| L-Alanine | b | Ala | HMDB0000161 | 56-41-7 | HY-N0229 | 90→44 | 23 | 20 | 10-1000 | 0.9999 |
| L-Arginine | m | Arg | HMDB0000517 | 74-79-3 | HY-N0455 | 175→70 | 38 | 23 | 50-1000 | 0.9906 |
| L-Asparagine | c | Asn | HMDB0000168 | 70-47-3 | HY-N0667 | 133→116 | 35 | 13 | 10-1000 | 0.9951 |
| L-Aspartic acid | d | Asp | HMDB0000191 | 56-84-8 | HY-N0666 | 134→74 | 40 | 17 | 10-1000 | 0.9973 |
| L-Cysteine | e | Cys | HMDB0000574 | 52-90-4 | HY-Y0337 | 122→76 | 30 | 17 | 10-1000 | 0.9981 |
| L-Glutamic acid | f | Glu | HMDB0000148 | 56-86-0 | HY-14608 | 148→102 | 86 | 15 | 50-1000 | 0.9975 |
| L-Histidine | n | His | HMDB0000177 | 71-00-1 | HY-N0832 | 156→110 | 30 | 20 | 50-1000 | 0.9941 |
| L-Isoleucine | o | Ile | HMDB0000172 | 73-32-5 | HY-N0771 | 132→86 | 30 | 14 | 10-1000 | 0.9901 |
| L-Leucine | g | Leu | HMDB0000687 | 61-90-5 | HY-N0486 | 132→30 | 40 | 20 | 20-1000 | 0.9912 |
| L-Lysine | p | Lys | HMDB0000182 | 56-87-1 | HY-N0469 | 147→84 | 30 | 23 | 50-1000 | 0.9965 |
| L-Methionine | q | Met | HMDB0000696 | 63-68-3 | HY-N0326 | 150→133 | 24 | 12 | 10-1000 | 0.9978 |
| L-Ornithine | h | Orn | HMDB0000214 | 70-26-8 | HY-B1352 | 133.1→70.3 | 41 | 20 | 10-1000 | 0.9959 |
| L-Phenylalanine | r | Phe | HMDB0000159 | 63-91-2 | HY-N0215 | 166→120 | 40 | 20 | 10-1000 | 0.9977 |
| L-Proline | s | Pro | HMDB0000162 | 147-85-3 | HY-Y0252 | 116→70 | 60 | 27 | 10-1000 | 0.9933 |
| L-Serine | i | Ser | HMDB0000187 | 56-45-1 | HY-N0650 | 106→60 | 27 | 15 | 10-1000 | 0.9906 |
| L-Threonine | j | Thr | HMDB0000167 | 72-19-5 | HY-N0658 | 120→74 | 30 | 14 | 10-1000 | 0.9981 |
| L-Tryptophan | k | Trp | HMDB0000929 | 73-22-3 | HY-N0623 | 205→146 | 23 | 23 | 20-1000 | 0.9935 |
| L-Tyrosine | l | Tyr | HMDB0000158 | 60-18-4 | HY-N0473 | 182→136 | 36 | 17 | 10-1000 | 0.9964 |
| L-Valine | t | Val | HMDB0000883 | 72-18-4 | HY-N0717 | 118→72 | 40 | 13 | 10-1000 | 0.9964 |

*MS no. corresponds to the chromatogram shown in Figure 1A, 1C. ^#^Catalog ID is for the corresponding amino acid purchased from MedChem Express (Monmouth Junction, NJ, USA). AA, amino acid; CE, collision energy; DP, declustering potential; MRM, multiple reaction monitoring; MS, mass spectrometry; nM, nanomole.

**Supplementary Table 2.** Mass spectrometry parameters of 20 isotope-labeled amino acids

| **Chemical** | **MS no.^*^** | **MRM transition** | **DP (V)** | **CE (V)** |
| --- | --- | --- | --- | --- |
| Glycine-^13^C_2_,^15^N | a’ | 79→32.1 | 40 | 20 |
| L-Alanine-^13^C_3_,^15^N | b’ | 94.1→47.1 | 40 | 30 |
| L-Arginine-^13^C_6_,^15^N_4_ | m' | 185.1→75.2 | 40 | 30 |
| L-Asparagine-^15^N_2_ | c’ | 135.1→75 | 80 | 30 |
| L-Aspartic acid-^13^C_4_,^15^N | d’ | 139.1→77 | 30 | 30 |
| L-Cystine-3,3,3′,3′-d_4_ | e’ | 245.2→122.2 | 30 | 25 |
| L-Glutamic acid-^13^C_5_,^15^N | f’ | 154→89.1 | 30 | 25 |
| L-Histidine-^15^N_3_ | n’ | 159→113 | 30 | 20 |
| L-Isoleucine-^13^C_6_,^15^N | o’ | 139→92.1 | 60 | 30 |
| L-Leucine-^13^C_6_,^15^N | g’ | 138.1→91 | 30 | 20 |
| L-Lysine-^13^C_6_,^15^N_2_ | p’ | 155.1→90.1 | 40 | 25 |
| L-Methionine-^15^N | q’ | 156→109.1 | 65 | 20 |
| L-Glutamine-^15^N_2_ | h’ | 149→131 | 40 | 15 |
| L-Phenylalanine-^13^C_9_,^15^N | r’ | 176.1→129 | 40 | 20 |
| L-Proline-^13^C_5_,^15^N | s’ | 122.1→75.1 | 30 | 20 |
| L-Serine-^13^C_3_,^15^N | i’ | 110.1→63 | 40 | 20 |
| L-Threonine-^13^C_4_,^15^N | j’ | 125.1→78.1 | 20 | 20 |
| L-Tryptophan-^15^N_2_ | k’ | 207.1→189.1 | 50 | 15 |
| L-Tyroxine-^13^C_9_,^15^N | l’ | 192.1→130.1 | 50 | 25 |
| L-Valine-^13^C_5_,^15^N | t’ | 124.1→77.1 | 25 | 20 |

*MS no. corresponds to the chromatogram shown in Figure 1B, 1D. MRM, multiple reaction monitoring; DP, declustering potential; CE, collision energy.

**Supplementary Table 3.** Gradient program of liquid chromatography for 20 amino acids

| **Total time (min)** | **Flow rate (μLmin)** | **A^a^ (v/v, %)** | **B^b^ (v/v, %)** |
| --- | --- | --- | --- |
| 0.00 | 300 | 99.0 | 1 |
| 1.50 | 300 | 98.0 | 2 |
| 2.50 | 300 | 80.0 | 20.0 |
| 3.00 | 300 | 5.0 | 95.0 |
| 4.50 | 300 | 5.0 | 95.0 |
| 5.00 | 300 | 99.0 | 1.0 |
| 8.00 | 300 | 99.0 | 1.0 |

^a^Solvent A, water containing 0.1% formic acid (v/v) and 0.05% trifluoroacetic acid; ^b^Solvent B, acetonitrile.

**Supplementary Table 4.** General characteristics of the study population

|  | **CON (n=112)** | **T2DM (n=101)** | **DKD (n=101)** | ***P1**** | ***P2**** |
| --- | --- | --- | --- | --- | --- |
| Age, years | 54.2±7.4 | 56.6±9.5 | 55.7±11.6 | 0.542 | 0.237 |
| Male/Female, n | 57/55 | 55/46 | 64/37 | 0.253 | 0.073 |
| Hb, g/L | 145.7±9.3 | 132.5±14.1 | 101.0±22.5 | <0.001 | <0.001 |
| Alb, g/L | 47.6±3.1 | 41.5±3.6 | 33.8±8.6 | <0.001 | <0.001 |
| SCr, μM | 68.1±10.5 | 62.4±14.8 | 359.6±280.6 | <0.001 | <0.001 |
| TC, mM | 4.3±0.6 | 4.1±1.3 | 5.2±1.7 | <0.001 | <0.001 |
| TG, mM | 1.1±0.4 | 1.8±1.5 | 1.8±0.9 | 0.625 | <0.001 |
| LDL-C, mM | 2.1±0.8 | 2.4±0.9 | 3.2±1.7 | 0.001 | <0.001 |
| HDL-C, mM | 2.1±0.8 | 1.1±1.0 | 1.2±0.6 | 0.906 | 0.009 |
| PTH, pg/mL | / | 29.8±11.7 | 111.8±87.9 | / | <0.001 |
| HbAlc, % | / | 7.2±1.4 | 9.0±2.2 | / | <0.001 |
| UAER, mg/24h | / | 16.6±13.9 | 2352.0±2388.9 | / | <0.001 |
| eGFR, mL/min/1.73m^2^ | 105.0±19.5 | 100.7±14.0 | 37.4±33.8 | <0.001 | <0.001 |
| Comorbidity |  |  |  |  |  |
| CAD, n | 0 | 22 | 29 | 0.331 | <0.001 |
| HTN, n | 0 | 47 | 84 | <0.001 | <0.001 |
| DR, n | 0 | 58 | 44 | 0.067 | <0.001 |
| DPN, n | 0 | 63 | 66 | 0.770 | <0.001 |

PTH, HbAlc, and UAER values were not tested for healthy subjects in regular health examination. Continuous variables are presented as mean ± standard deviation and were compared with the Student’s t test. Categorical variables were compared by Chi-square test or Fisher’s exact test and presented as counts. *P1*: DKD vs T2DM group; *P2*: DKD vs CON group. Alb, albumin, CAD, coronary artery disease; CON, healthy subjects; DKD, diabetic kidney disease; DPN, diabetic peripheral neuropathy; DR, diabetic retinopathy; eGFR, estimated glomerular filtration rate; Hb, hemoglobin; HbAlc, glycated hemoglobin; HDL-C, high density lipoprotein cholesterol; LDL-C, low density lipoprotein cholesterol; NA, not available; PTH, parathyroid hormone; Scr, serum creatinine; T2DM, type 2 diabetes mellitus; TC, total cholesterol; TG, triglyceride; UAER, urinary albumin excretion rate.

**Supplementary Table 5.** Plasma levels of 20 amino acids in diabetic kidney disease patients with different levels of kidney function

| **Amino acid**  **(μM)** | **CKD 1**  **(n=13)** | **CKD 2-3**  **(n=30)** | **CKD 4-5**  **(n=58)** | **CKD 2-3 *vs* CKD 1**  ***P* value** | **CKD 4-5 *vs* CKD 2-3**  ***P* value** |
| --- | --- | --- | --- | --- | --- |
| Glycine | 284.0±119.5 (128.8-517.8) | 313.9±188.3 (119.1-932.1) | 160.6±115.0 (14.87-598.1) | 0.602 | <0.001 |
| L-Alanine | 228.2±77.0 (114.8-344.8) | 180.7±41.8 (110.2-267.8) | 77.1±52.3 (18.3-235.5) | 0.012 | <0.001 |
| L-Arginine | 248.9±89.0 (100.8-433.7) | 272.1±175.3 (107.8±889.0) | 243.5±102.7 (80.6-546.9) | 0.655 | 0.337 |
| L-Asparagine | 35.6±10.3 (21.2-56.6) | 30.5±13.9 (9.1-68.4) | 11.4±11.6 (1.4-69.0) | 0.238 | <0.001 |
| L-Aspartic acid | 52.9±64.3 (0.0-192.9) | 61.2±53.1 (1.8-184.8) | 50.7±70.2 (3.6-325.4) | 0.658 | 0.471 |
| L-Cysteine | 245.2±74.7(147.1-382.0) | 298.1±163.6 (126.1-894.4) | 465.0±218.9 (0.0-1056) | 0.273 | <0.001 |
| L-Glutamic acid | 208.3±80.8 (104.5-361.0) | 195.8±72.2 (74.0-411.3) | 214.1±101.6 (112.4-738.1) | 0.619 | 0.383 |
| L-Histidine | 24.5±14.5 (8.3-60.3) | 22.4±17.6 (9.5-107.8) | 9.7±8.1 (1.5-48.3) | 0.716 | <0.001 |
| L-Isoleucine | 97.6±30.3 (63.6-163.8) | 75.6±12.7 (52.5-105.7) | 40.92±16.6 (13.9-83.0) | 0.002 | <0.001 |
| L-Leucine | 180.5±58.3 (105.0-297.4) | 135.9±22.4 (101.2-176.7) | 51.2±38.2 (10.7-142.2) | <0.001 | <0.001 |
| L-Lysine | 166.6±45.2 (110.7-274.8) | 129.9±29.6 (85.1-191.3) | 26.3±52.6 (5.9-182.9) | 0.003 | <0.001 |
| L-Methionine | 69.9±24.3 (50.2-143.3) | 58.4±11.7 (35.0-78.7) | 39.4±14.0 (14.4-92.7) | 0.041 | <0.001 |
| L-Ornithine | 52.8±20.6 (27.1-91.1) | 47.5±22.1 (26.7-152.5) | 22.8±14.1 (3.1-85.1) | 0.469 | <0.001 |
| L-Phenylalanine | 91.9±11.5 (76.9-113.1) | 83.5±16.3 (58.1-127.2) | 49.9±18.4 (24.5-94.7) | 0.101 | <0.001 |
| L-Proline | 168.9±49.8 (78.0-274.8) | 168.9±79.9 (67.1-407.3) | 59.8±57.9 (7.8-244.6) | 0.999 | <0.001 |
| L-Serine | 64.1±23.1 (41.3-108.3) | 56.7±24.5 (28.0-134.2) | 25.6±18.5 (5.4-77.2) | 0.360 | <0.001 |
| L-Threonine | 96.6±27.8 (65.2-177.8) | 82.6±33.1 (27.1-190.2) | 30.3±28.0 (4.3-162.2) | 0.189 | <0.001 |
| L-Tryptophan | 67.9±20.7 (36.9-108.0) | 46.4±18.9 (2.1-92.7) | 60.8±40.0 (1.8-211.2) | 0.002 | 0.066 |
| L-Tyrosine | 50.3±17.4 (23.2-77.6) | 36.7±8.4 (23.8-62.5) | 25.7±11.4 (10.2-79.4) | 0.001 | <0.001 |
| L-Valine | 134.9±17.6 (109.9-170.3) | 88.4±10.8 (70.7-106.9) | 24.1±24.5 (2.2-69.7) | <0.001 | <0.001 |

Data represent mean ± standard deviation (range). μM, micromole; CON, healthy controls; T2DM, type 2 diabetes mellitus; DKD, diabetic kidney disease.

**Supplementary Table 6.** Area under the receiver operating characteristic curve values for 20 amino acids in plasma of study participants

| **Amino acid** | **DKD vs T2DM** | |  | **DKD vs CON** | |  |
| --- | --- | --- | --- | --- | --- | --- |
|  | **AUC** | ***P* value*** |  | **AUC** | ***P* value*** |  |
| Glycine | 0.555±0.041 | 0.1742 |  | 0.660±0.038 | < 0.001 |  |
| L-Alanine | 0.806±0.030 | < 0.001 |  | 0.873±0.024 | < 0.001 |  |
| L-Arginine | 0.372±0.039 | 0.0016 |  | 0.459±0.042 | 0.3016 |  |
| L-Asparagine | 0.672±0.038 | < 0.001 |  | 0.827±0.027 | < 0.001 |  |
| L-Aspartic acid | 0.265±0.035 | < 0.001 |  | 0.451±0.040 | 0.2178 |  |
| L-Cysteine | 0.189±0.030 | < 0.001 |  | 0.341±0.037 | < 0.001 |  |
| L-Glutamic acid | 0.698±0.037 | < 0.001 |  | 0.511±0.041 | 0.788 |  |
| L-Histidine | 0.681±0.038 | < 0.001 |  | 0.993±0.006 | < 0.001 |  |
| L-Isoleucine | 0.819±0.030 | < 0.001 |  | 0.743±0.035 | < 0.001 |  |
| L-Leucine | 0.819±0.030 | < 0.001 |  | 0.775±0.033 | < 0.001 |  |
| L-Lysine | 0.658±0.039 | < 0.001 |  | 0.713±0.036 | < 0.001 |  |
| L-Methionine | 0.591±0.040 | 0.0249 |  | 0.677±0.037 | < 0.001 |  |
| L-Ornithine | 0.570±0.041 | 0.0863 |  | 0.763±0.032 | < 0.001 |  |
| L-Phenylalanine | 0.585±0.042 | 0.0362 |  | 0.726±0.035 | < 0.001 |  |
| L-Proline | 0.658±0.040 | < 0.001 |  | 0.697±0.038 | < 0.001 |  |
| L-Serine | 0.696±0.037 | < 0.001 |  | 0.810±0.029 | < 0.001 |  |
| L-Threonine | 0.723±0.035 | < 0.001 |  | 0.734±0.035 | < 0.001 |  |
| L-Tryptophan | 0.709±0.038 | < 0.001 |  | 0.740±0.035 | < 0.001 |  |
| L-Tyrosine | 0.835±0.030 | < 0.001 |  | 0.864±0.026 | < 0.001 |  |
| L-Valine | 0.874±0.025 | < 0.001 |  | 0.881±0.023 | < 0.001 |  |

Data represent mean ± standard deviation. **P* values were determined by receiver operating characteristic analysis under nonparametric assumption. AUC, area under the receiver operating characteristic curve; CON, healthy subjects; DKD, diabetic kidney disease; T2DM, type 2 diabetes mellitus;.


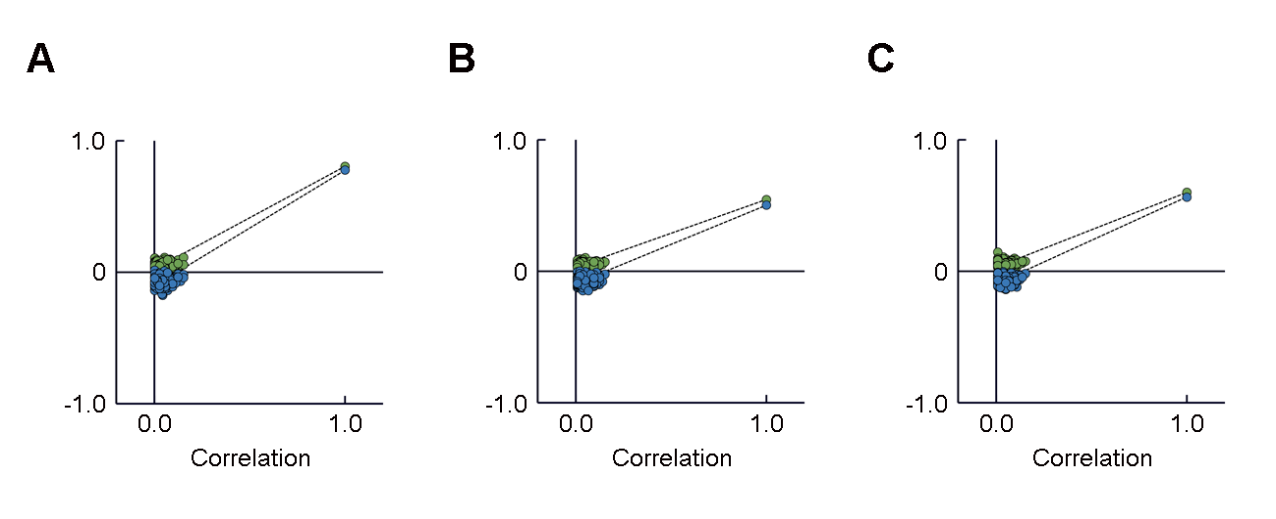


**Supplementary Figure 1. Permutation plots of orthogonal partial least-squares discriminant (OPLS-DA) models.** Validity and degree of overfitting in the OPLS-DA model of **(A)** healthy subjects, **(B)** T2DM patients, and **(C)** DKD patients. The permutation indicates the correlation coefficient between the original R^2^ (green dots), Q^2^ (blue dots), and cumulative R^2^, Y^2^; the dashed lines represent the corresponding regression lines. The number of random permutation tests for each plot was 200.
